# A Comprehensive View on the Impact of Chlorogenic Acids on Colorectal Cancer

**DOI:** 10.3390/cimb46070405

**Published:** 2024-07-02

**Authors:** Andreea-Adriana Neamțu, Teodor Andrei Maghiar, Violeta Turcuș, Paula Bianca Maghiar, Anca-Maria Căpraru, Bianca-Andreea Lazar, Cristina-Adriana Dehelean, Ovidiu Laurean Pop, Carmen Neamțu, Bogdan Dan Totolici, Endre Mathe

**Affiliations:** 1 Department of Toxicology, “Victor Babes” University of Medicine and Pharmacy, Eftimie Murgu Square, No. 2, 300041 Timisoara, Romania; aneamtu94@gmail.com (A.-A.N.); cadehelean@umft.ro (C.-A.D.); 2 Research Centre for Pharmaco-Toxicological Evaluation, “Victor Babes” University of Medicine and Pharmacy, Eftimie Murgu Square, No. 2, 300041 Timisoara, Romania; 3 Clinical County Emergency Hospital of Arad, Andrenyi Karoly Str., No. 2-4, 310037 Arad, Romania; totolici.bogdan@uvvg.ro; 4 Clinical County Hospital of Târgu Mureș, 1 Decembrie 1918 Blvd., No. 1, 540011 Târgu Mures, Romania; mitran.anca@hotmail.com (A.-M.C.); ohii.bianca@yahoo.com (B.-A.L.); 5 Doctoral School of Biomedical Sciences, University of Oradea, Universității Str., No. 1, 410087 Oradea, Romania; teodormaghiar@yahoo.com (T.A.M.); pao.badea@gmail.com (P.B.M.); 6 Clinical County Emergency Hospital of Oradea, Gheorghe Doja Str., No. 65, 410169 Oradea, Romania; 7 Pelican Hospital, Corneliu Coposu Str., No. 2, 410450 Oradea, Romania; 8 Faculty of Medicine and Faculty of Dentistry, “Vasile Goldis” Western University of Arad, Liviu Rebreanu Str., No. 86, 310045 Arad, Romania; endre.mathe@agr.unideb.hu; 9 National Institute for Economic Research “Costin C. Kiritescu” of the Romanian Academy/Centre for Mountain Economy (CE-MONT), 725700 Suceava, Romania; 10 Poiana Mare Psychiatry Hospital, Gării Str., No. 40, 207470 Poiana Mare, Romania; 11 Faculty of Medicine and Pharmacy, University of Oradea, Universității Str., No. 1, 410081 Oradea, Romania; drovipop@yahoo.com; 12 Institute of Nutrition, Faculty of Agricultural and Food Sciences and Environmental Management, University of Debrecen, Böszörményi Str., No. 138, H-4032 Debrecen, Hungary

**Keywords:** chlorogenic acids, caffeoylquinic acids, hydrocoumaric acids, colorectal cancer

## Abstract

Chlorogenic acids are plant secondary metabolites, chemically—polyphenols with similar biological activity, formed through the esterification of quinic acid and hydrocinnamic acid moieties. They are best known for their high concentration in coffee and other dietary sources and the antioxidant properties that they exhibit. Both chlorogenic acids and plant extracts containing significant amounts of the compounds show promising in vitro activity against colorectal cancer. With coffee being the most popular drink in the world, and colorectal cancer at an unfortunate peak in incidence and mortality, the mechanisms through which the anti-tumorigenic effect of chlorogenic acids could be functionalized for CRC prevention seem appealing to study. Therefore, this review aims to enable a better understanding of the modes of action of chlorogenic acids in combating carcinogenesis, with a focus on cell cycle arrest, the induction of apoptosis, and the modulation of Wnt, Pi3K/Akt, and MAPK signal transduction pathways, alongside the reduction in the number of inflammatory cytokines and chemokines and the counterintuitive beneficial elevation of oxidative stress.

## 1. Introduction

Currently, colorectal cancer (CRC) is the subject of scientific and public health inquiries of critical importance. It represents a significant health issue globally, ranking third in incidence and second in mortality among all cancers [1]. Its relevance is underscored by the disease’s complexity, alongside its impact on patient quality of life and healthcare systems. Recognizing the seriousness of this disease, it has become imperative to understand the etiology of the malignancy and consider different types of preventive action. Environmental factors, particularly nutrition, play a substantial role in colorectal carcinogenesis [2]. It is estimated that about 70–90% of CRC cases can be attributed to diet and lifestyle [3]. The strong influence of modifiable risk factors distinguishes CRC from many other types of cancer with a higher genetic predisposition, deeming it more suitable for primary preventive strategies [4].

Research shows that diets high in red and processed meats, low in fiber, and rich in alcohol consumption are associated with an increased risk of CRC development [5,6,7,8]. Conversely, diets rich in fruits, vegetables, and whole grains are linked to a reduced risk [9,10,11,12]. This direct correlation between diet and CRC risk highlights the potential for nutrition-focused preventive strategies.

There are numerous studies that support coffee consumption for the prevention of CRC development [13,14], improved prognosis, or decreased chances of recurrence [15,16]. As the most widely consumed beverage in the world [17,18], it is pleasing to observe coffee’s beneficial effects on health. Potential mechanisms suggesting how the consumption of coffee could impact the development of colorectal cancer center on the diverse chemopreventive qualities inherent to the various components of coffee, acting in a synergistic manner [19]. These protective qualities span a broad spectrum, encompassing anti-inflammatory, antioxidant, and antiproliferative properties, alongside pro-apoptotic effects [20]. Furthermore, during digestion, host enzymes and the gut microbiome interact with compounds in coffee, leading to the generation of bioactive metabolites that possess chemopreventive characteristics [21].

However, in order to formulate accurate scientific evidence-based dietary recommendations, it is necessary to analyze the intricate activity of individual compounds, beyond general dietary patterns. Nutrients, much like pharmacological agents, can modulate critical cellular signaling pathways, impacting the initiation, progression, and potentially the treatment of CRC [17,22,23,24,25,26,27,28].

Chlorogenic acids represent plant secondary metabolites and are chemically polyphenols, formed through the esterification of quinic acid and hydrocinnamic acid moieties. They are best-known for their high concentration in coffee and other dietary sources and the antioxidant properties that they exhibit [29].

## 2. Methods—Literature Search Methodology

The current review is divided into two main approaches—a narrative review regarding chlorogenic acids (Section 3) and a systematic review that is focused on their mechanisms of action in colorectal cancer (Section 4). The literature search methodology is specific to each section. The database surveyed for all the information included in this article is PubMed.

### 2.1. Narrative Review (Section 3)

Section 3 is written as a narrative review. It is structured so that a comprehensive background on chlorogenic acids is provided. The search was conducted using keywords belonging to the section title AND “chlorogenic acids” in the PubMed database. Articles in languages other than English were excluded. Relevant articles were included, despite the article type. The search was not exhaustive; however, it was aimed at choosing the most relevant and recent sources of information.

### 2.2. Systemativ Review (Section 4)

**Figure 1:**
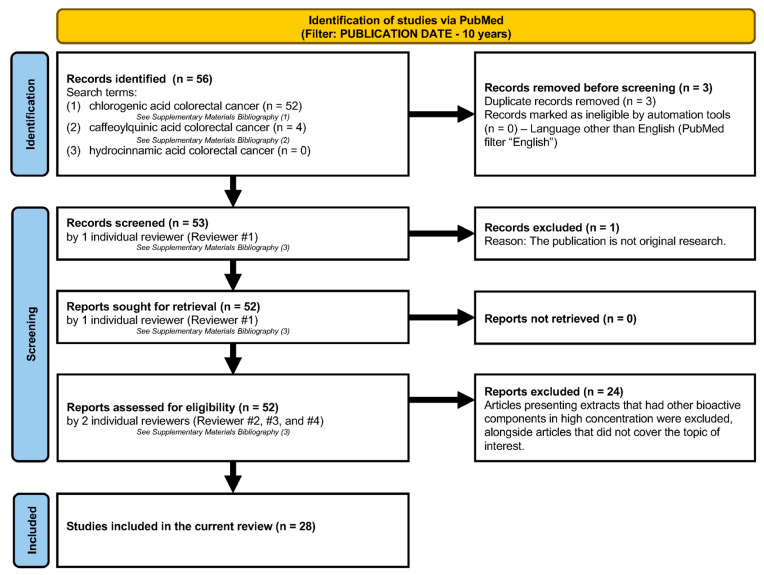
Search methodology for the systematic review of literature.

Section 4 is written as a systematic review that follows the PRISMA 2020 guidelines (Figure 1). The information presented in Section 4 was collected by surveying the PubMed database with the search terms “chlorogenic acid colorectal cancer”, “caffeoylquinic acid colorectal cancer”, “hydrocinnamic acid colorectal cancer” included in the title and abstract in the past 10 years (2014–2024), exclusively including original articles (without reviews and meta-analysis) published in English. The identification, initial screening, and retrieval were conducted by one of our reviewers, while the assessment for eligibility was conducted individually by three investigators in our team. Articles presenting extracts that had other bioactive components in high concentrations were excluded, alongside articles that did not cover the topic of interest. The data were then divided into appropriate chapters and summarized.

## 3. Background Information on Chlorogenic Acids

### 3.1. Chemical Characteristics of Chlorogenic Acids

**Figure 2:**
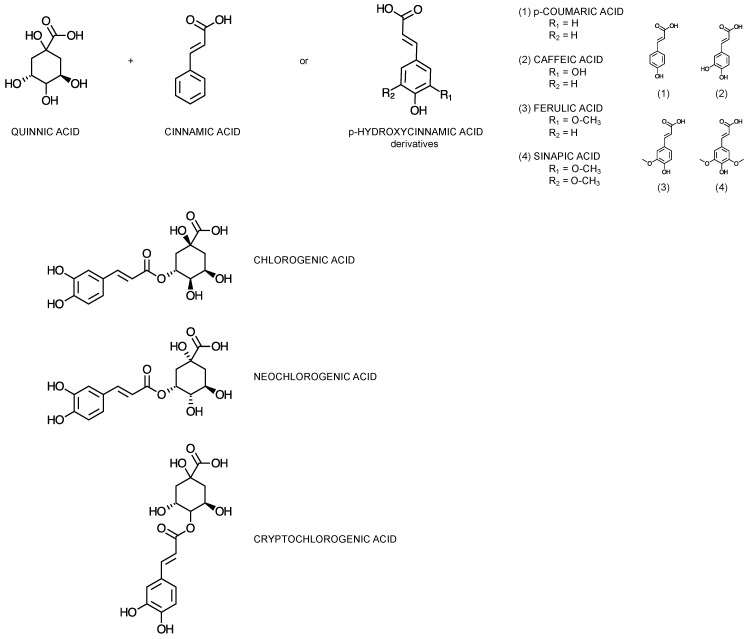
Chemical structures of the subunits forming chlorogenic acids, and the most abundant subclass representatives: 5-*O*-caffeoylquinic acid (chlorogenic acid), 3-*O*-caffeoylquinic acid (neochlorogenic acid), and 4-*O*-caffeoylquinic acid (cryptochlorogenic acid).

Chlorogenic acids (CGAs), interchangeably also referred to as caffeoylquinic acids [30], represent a significant subclass of phenolic compounds [31]. They are formed through the esterification of quinic acid derivatives with one to four residues of hydrocinnamic or trans-cinnamic acids (Figure 2), such as caffeic, p-coumaric, ferulic and sinapic acid [32,33]. The most commonly encountered members of the subclass are the caffeoylquinic acid (CQA) isomers in positions 5, 3, and 4, namely 5-*O*-caffeoylquinic acid (5-CQA), also known as chlorogenic acid, 3-*O*-caffeoylquinic acid (3-CQA), also known as neochlorogenic acid, and 4-*O*-caffeoylquinic acid (4-CQA), also known as cryptochlorogenic acid, respectively [34].

**Figure 3:**
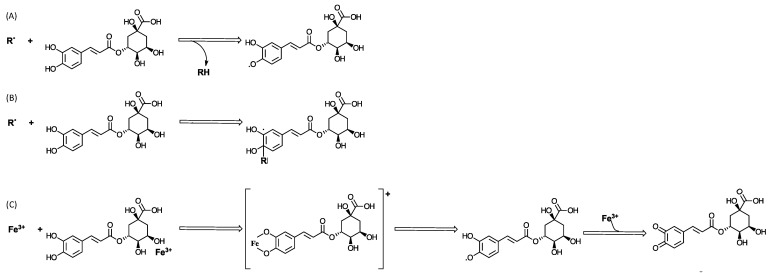
Hypothesized mechanisms of reactive oxygen species (ROS) scavenging employed by CGAs. Direct mechanisms: (**A**) hydrogen atom transfer (HAT); (**B**) radical adduct formation (RAF). Indirect mechanism: (**C**) metal chelation.

From a chemical perspective, CGAs have demonstrated significant reactive oxygen species (ROS) scavenging potential through two direct mechanisms: hydrogen atom transfer (HAT) and radical adduct formation (RAF) [35]. In the HAT mechanism (Figure 3A), free radicals abstract a hydrogen atom from the catechol moiety in CQAs, which effectively neutralizes the free radicals and prevents them from causing oxidative damage [36,37]. This reaction showcases the role of chlorogenic acid as a hydrogen donor, an essential characteristic of antioxidants. Alternatively, the RAF mechanism (Figure 3B) involves the addition of a free radical to chlorogenic acid, resulting in the formation of a stable radical intermediate [35,37]. This process effectively quenches the reactive and potentially damaging free radicals, inhibiting further oxidation processes. Additionally, CGAs exhibit an indirect antioxidant mechanism through metal chelation [35,38,39]. It reacts with redox-active transition metals (Figure 3C), such as iron [39] and copper [38], which are known to catalyze the generation of reactive oxygen species. By binding to these metals, CGAs form stable complexes through their hydroxycinnamic moiety, leading to the formation of corresponding quinones [37]. This action prevents the metals from participating in oxidative reactions, further contributing to the compounds’ overall antioxidant efficacy.

CGAs undergo degradation upon exposure to heat, notably during roasting processes [34]. There is a paucity of research regarding the direct formation of volatile compounds from chlorogenic acids [40]. Several studies document the formation of chlorogenic acids’ isomerization products upon heat exposure [34,40,41,42,43], followed by lactone formation, caused by the loss of one water molecule from the quinic acid moiety [44]. Moreover, carbon–carbon bond breakage, caused by exposure to high temperatures, has also been documented and hypothesized to lead to the formation of volatile compounds [41].

In revising the literature, it is extremely important to carefully assess whether the described compounds follow the current (post-1976) International Union of Pure and Applied Chemistry (IUPAC) nomenclature or the outdated convention [45]. It is often the case that scientific articles still describe 5-*O*-caffeoylquinic acid (5-CQA), commonly referred to as chlorogenic acid, one of the most abundant CGAs, as 3-*O*-caffeoylquinic acid [31]. In this work, the current IUPAC nomenclature is used.

### 3.2. Dietary Sources of Chlorogenic Acids

CGAs represent key bioactive compounds with notable health benefits [46], rendering them an integral component of a balanced diet. They are ubiquitously found in a diverse array of dietary sources. Notably abundant in coffee, CGAs’ concentration is greatly influenced by the type and degree of roasting, alongside the type of coffee beans, with a higher level in the Robusta (*Coffea canephora*) as compared to the Arabica (*Coffea arabica*) variety [47,48]. Green (unroasted) coffee beans are especially rich in CGAs, containing up to approximately 150 mg/g of dry weight [48,49,50,51], while the concentration in roasted coffee can vary widely, typically ranging from 20 to 75 mg/g depending on the roast degree [42]; lighter roasts generally retain more CGAs than darker roasts [47]. The predominant CGAs in green coffee are 5-caffeoylquinic acid (5-CQA), which comprises about 50–60% of the total CGA content [47,52], and 3-caffeoylquinic acid (3-CQA) and 4-caffeoylquinic acid (4-CQA), each constituting roughly 10–15% [52]. Additionally, feruloylquinic and dicaffeoylquinic acids are present in smaller amounts, making up the remaining 10–20% [52]. Beyond coffee, other brews such as yerba mate (*Ilex paraguariensis*), typically prepared as an infusion, can also contribute to CGA intake, providing about 80–90 mg/g of dry weight [53,54].

Albeit in lower quantities, vegetables and fruits are also dietary sources of CGAs. Artichokes, probably the vegetable richest in CGAs, contain up to 80 mg/g CGAs as a mixture of mono- and dicaffeoylquinic acids [55,56]. Eggplant has been proven to contain up to 28 mg/g 5-CQA, representing 80–95% of the total CGAs [46,57]. Carrots also rank highly based on their CGA content of up to 18.9 mg/g [46,58]. As a popular side dish, potatoes also present a concentration of CGAs of up to 3 mg/g [46,59].

Despite having a generally lower content of CGAs, fruits offer a different chemical profile. Apples, providing up to 2 mg/g of CGAs, mainly contain 3-caffeoylquinic acid, 5-caffeoylquinic acid, and 4,5-dicaffeoylquinic acid [46,56,60,61]. Blueberries stand out for their high content of CGAs, around 2.0 mg/g, boasting a rich array including 3-caffeoyl, 4-caffeoyl, and 5-caffeoylquinic acids, as well as feruloylquinic and isoferuloylquinic acids [46,56,62,63].

The diversity of sources for the uptake of CGAs, from various brews to vegetables and fruits, highlights their accessibility in a balanced diet and underscores their importance in nutrition and health. The concentration of CGAs in these dietary sources can be influenced by various factors, including the method of food processing and preparation. This comprehensive knowledge aids in guiding dietary recommendations and enhancing public health initiatives focused on leveraging the benefits of CGAs as nutraceuticals and natural antioxidants.

### 3.3. Chlorogenic Acids Biosynthesis in Plants

CGAs, as plant secondary metabolites, play a critical role in plant defense and survival strategies. These compounds not only afford protection against oxidative stress and pathogens, but also aid in wound repair. As secondary metabolites, CGAs are integral in bolstering a plant’s resilience to environmental challenges [64]. Beyond these defensive roles, chlorogenic acids also influence plant growth and development and participate in the formation of pigments that are crucial for attracting pollinators and following up with the pollination processes [65]. Thus, CGAs are vital to the health, adaptability, and survival of plants in their diverse habitats.

The biosynthesis of CGAs is intricate, sometimes offering alternative mechanisms. Although the biosynthesis of 5-CQA is well documented, the pathways leading from 5-CQA to other CQAs are less clear [66]. It is generally believed that other CQAs are derived from 5-CQA, but the specific isomerases involved in these conversions are not well characterized. Furthermore, the biosynthesis of CGAs with substituents other than caffeic acid remains largely unexplored [67].

**Figure 4:**
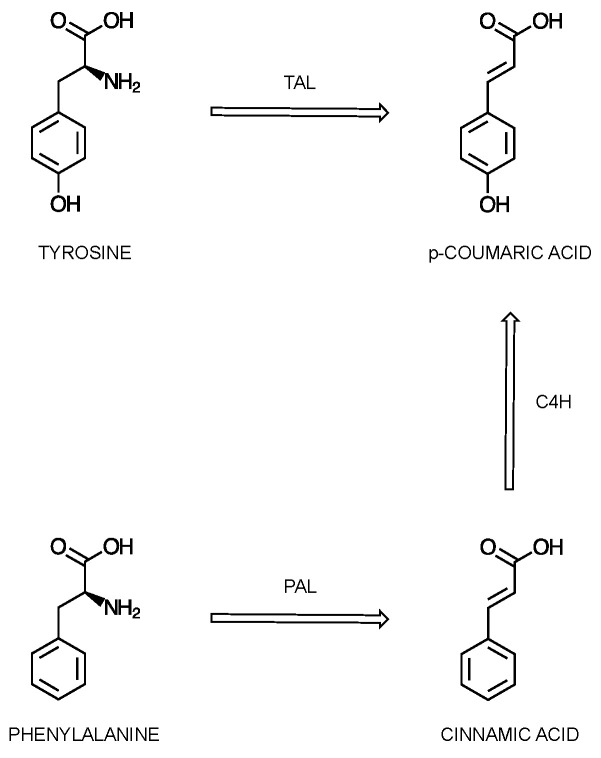
Proposed scheme of the initial step in the biosynthesis of chlorogenic acids, using phenylalanine (Phe) and tyrosine (Tyr) as substrates, which are initially transformed into *p*-coumaric acid.

Specifically, CGA synthesis starts with the phenylpropanoid pathway [66]. The first step takes place in the cytosol, using one of two routes [67]. The initial substrates are either phenylalanine (Phe) or tyrosine (Tyr), which are initially transformed into *p*-coumaric acid (Figure 4). The amino acids are initially transformed into cinnamic acid (for Phe) and *p*-coumaric acid (for Tyr), under the action of Phe ammonia lyase (PAL) and Tyr ammonia lyase (TAL), respectively [66,67,68]. In the case of Phe, an additional reaction is necessary for the transformation of cinnamate into *p*-coumaric acid. This reaction is catalyzed by the enzyme cinnamate 4-hydroxylase (C4H). PAL, catalyzing the initial step, plays a critical role in directing the flow of molecules from the primary metabolism into the secondary metabolism for the biosynthesis of CQAs, with its regulation occurring at multiple levels and thoroughly affecting the rate of the reaction [66,69].

**Figure 5:**
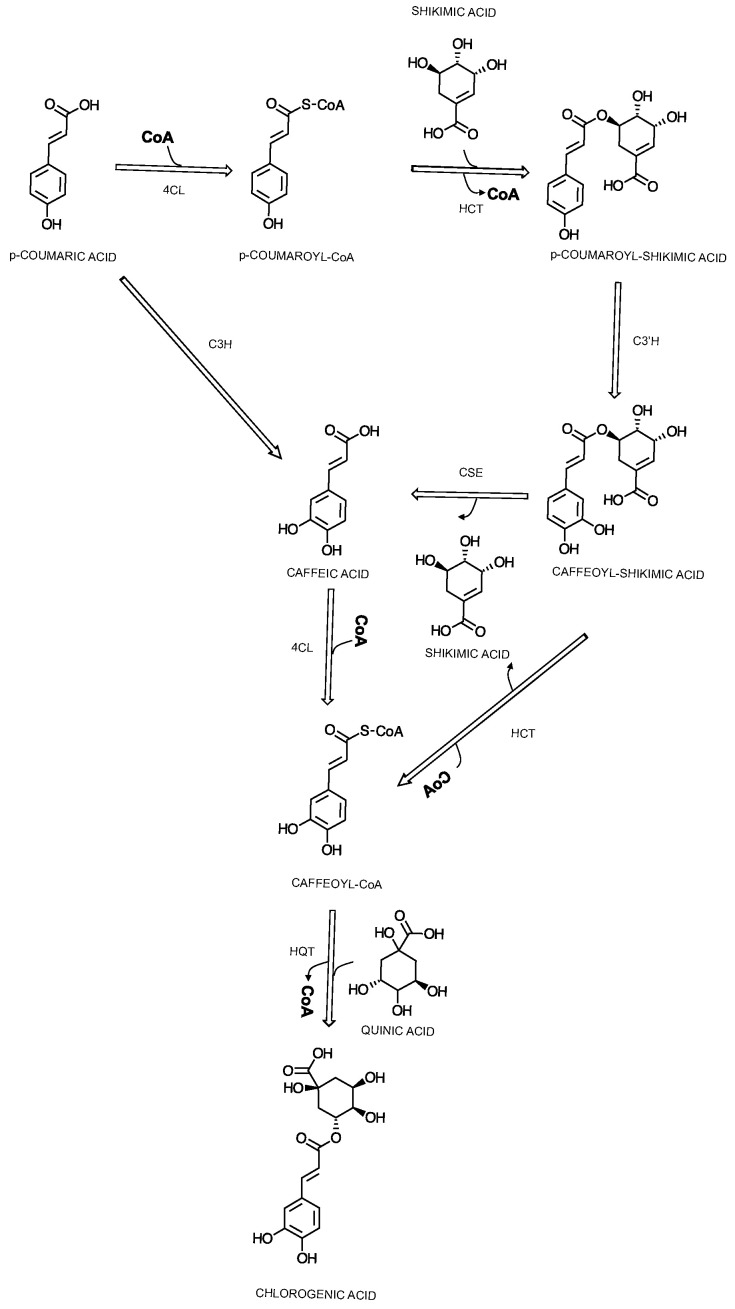
Proposed scheme of the biosynthesis of chlorogenic acids, focused on 5-caffeoylquinic acid, through shikimic acid metabolism.

**Figure 6:**
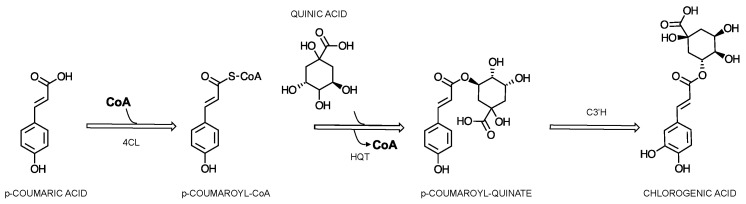
Proposed scheme of the biosynthesis of chlorogenic acids, focused on 5-caffeoylquinic acid, through quinic acid metabolism.

From here, two alternative pathways could be available. The *p*-coumaric acid can either be turned into *p*-coumaroyl-CoA by 4-cinnamoyl-CoA ligase (4CL) or directly into caffeic acid by the cytochrome P450 oxidase *p*-coumaroyl-3′-hydroxylase (C3H) [67]. This compound, *p*-coumaroyl-CoA, stands at a crucial junction, participating in various pathways including the synthesis of flavonoids, stilbenes, and monolignols. The specific localization of 4CL isoforms within the cell is instrumental in determining whether the biosynthetic pathway will produce *p*-coumaroyl-CoA or diverge to other compounds in the phenylpropanoid pathway. Two alternative pathways can be adopted from here for the synthesis of CGAs, namely the shikimic acid metabolism (Figure 5) or the quinic acid metabolism pathway (Figure 6). The former would lead to the formation of caffeic acid through the involvement of hydroxycinnamoyl-CoA:shikimic acid/quinic acid hydroxycinnamoyl-transferase (HCT) [66], cytochrome P450 *p*-coumaric acid ester-specific 3′-hydroxlase (C3′H), and caffeoyl shikimic acid esterase (CSE) and the formation of p-coumaroylshikimic acid, caffeoylshikimic acid, and caffeic acid, respectively [67], as an alternative to the reaction catalyzed by C3H. From here, the involvement of three other enzymes, 4CL, HCT and its alternative, hydroxycinnamoyl-CoA, and quinic acid hydroxycinnamoyltransferase (HQT) leads to the formation of 5-CQA [66,67,68,69]. The latter pathway, through quinic acid metabolism (Figure 6), leads to the formation of 5-CQA through *p*-coumaroylquinic acid, the first reaction being catalyzed by HQT, followed by C3’H [66,67].

### 3.4. Chlorogenic Acids Bioavailability in Humans

**Figure 7:**
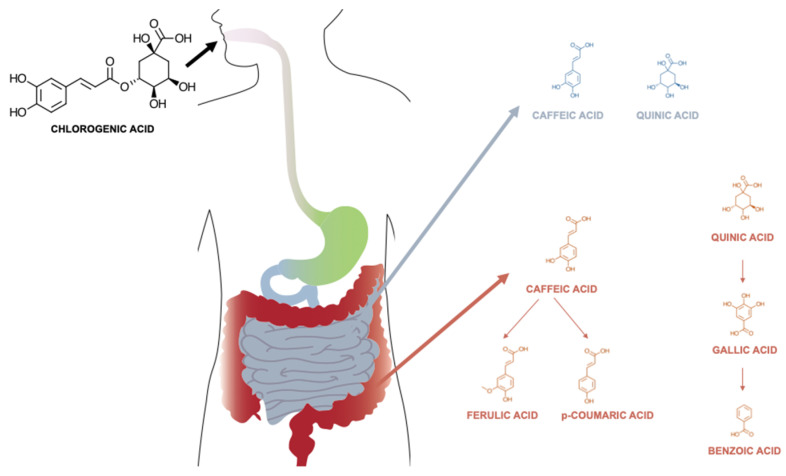
Proposed scheme of the bioavailability (digestion, absorption and metabolism) of chlorogenic acids, focused on 5-caffeoylquinic acid.

The bioavailability of CGAs in humans involves a complex process of digestion, absorption, and metabolism (Figure 7), primarily occurring within the stomach, small and large intestines, governed by the interplay between host enzymes and gut microbiota [59,62,63].

In the stomach and small intestine, CGAs undergo initial digestion and absorption [70,71,72,73]. Both in vivo and in vitro studies point out the possibility of the direct absorption of some CGAs [72,73], alongside the possibility of digestion through hydrolysis, followed by absorption [70,71]. Direct absorption was observed in research studies conducted with cultured gastric epithelial cells and demonstrated that CQAs, FQAs, and caffeoylquinolactones (CQLs) are capable of crossing the epithelial barrier [74]. It is suggested that the 3- and 5-regio-isomers of these compounds likely traverse the epithelium through passive diffusion, primarily via the paracellular route. In contrast, for compounds like 4-CQA and 4-FQA, a facilitated transport mechanism is believed to play a role, suggesting a more selective and efficient mode of absorption. Dicaffeoylquinic acids (DiCQAs) have been observed to pass through cellular membranes at an even faster rate. This increased permeability is likely due to their higher hydrophobicity, which facilitates easier traversal across lipid bilayers. Specifically, in the case of 3,5-diCQA, there is evidence to suggest that its movement across the membrane is not just a passive process but also involves carrier-mediated efflux [68,74,75]. Digestion followed by absorption depends, however, on the presence of key enzymes on the mucosal surface, namely as mucosal esterases [67,68]. These enzymes hydrolyze CGAs to release hydrocinnamic acid and quinic acid, which are smaller molecules that are more readily absorbed [76]. However, the small intestine’s role in the absorption of CGAs is somewhat limited due to these compounds’ relatively low stability and poor solubility in the gastrointestinal environment [70,71].

About two-thirds of the total CGA content reaches the colon [77,78], where the involvement of the gut microbiota becomes more pronounced. The resident bacterial population uses bacterial enzymes, such as feruloyl esterases, beta-glucosidases, and demethylases, to further break down CGAs into various metabolites, including phenolic acids like ferulic acid and dihydroferulic acid [79]. This biotransformation by the gut bacteria enhances the bioavailability of CGAs, as these simpler metabolites are more easily absorbed by the lining cells of the colon.

Once absorbed, CGAs and their metabolites undergo extensive functionalization and conjugation, a process crucial for their biological activity and elimination.

## 4. Chlorogenic Acids—Mechanisms of Action in Colorectal Cancer: Review Methodology

CGAs have been the subject of numerous studies investigating their potential therapeutic effects in colorectal cancer [80,81,82,83,84,85,86,87,88,89,90,91,92,93,94,95,96,97,98,99,100,101,102,103,104,105,106,107]. Their mechanisms of action encompass a range of cellular processes, including cell cycle arrest (see Section 4.2), apoptosis induction (see Section 4.3), the migration and invasion (see Section 4.4) of cancer cells, and the modulation of key signaling pathways (see Section 4.5, Section 4.6 and Section 4.7), alongside their roles in oxidative stress (see Section 4.8) and inflammation management (see Section 4.9).

### 4.1. Chlorogenic Acids Impact Cell Viability in Colorectal Cancer Models

Most studies analyzed tested cell viability in colorectal cancer cell lines by employing the colorimetric viability assay MTT (Table 1). Despite being considered the “gold standard” for assessing cytotoxicity, its accuracy is often disputed [108], especially concerning experimental setups that involve cancer cells [109]. However, most studies perform follow ups with additional assessment of the activity of the compounds; therefore, it is reliable to say that chlorogenic acids reduce colon cancer cells’ viability in all types of analyzed cell cultures, namely HT-29, SW480, SW620, HCT116, Caco-2, CT26, RKO, and the tumor cells of Sprague Dawley AOM-induced rats.

### 4.2. Chlorogenic Acids Impact the Cell Cycle in Colorectal Cancer Models

The research on colorectal cancer cells primarily focuses on how the regulation deviates from physiology (Table 2). Several key enzymes, such as cyclins, cyclin-dependent kinases (CDKs), CDK inhibitors, and the p53 protein represent the main focus [110]. Diverse patterns in the expression of cell cycle regulators among different colorectal cancer cell lines are usually observed [80,83,91,107]. This variation suggests a link to the unique biological characteristics of the cancer cells and their specific genetic origins. Alternatively, the differential regulation might point out different influences of the substances tested.

A notable finding is the relationship between mutations in the p53 gene and the expression of p21, a protein involved in cell cycle regulation. Typically, mutations in the p53 gene lead to reduced p21 expression [110]. However, p21 can still be activated through pathways that are both dependent and independent of the p53 protein in colorectal cancer.

Furthermore, an increased expression of D-type cyclins, particularly D2 and D3, is observed in most of the cell lines [101]. This overexpression, coupled with the loss of p16 protein, leads to an increase in the content of CDKs. In the studies analyzed, the quantifications focus on cyclin-D1 content [81,83,107], which is notably decreased upon chlorogenic acid treatment.

A number of studies focus on the phase of cell cycle arrest [82,83,87,88,89,95,104,107], demonstrating the effect of chlorogenic acids at a cellular level.

### 4.3. Chlorogenic Acids Impact Apoptosis in Colorectal Cancer Models

The central element in CRC evasion of apoptosis is the β-catenin/T-cell factor (Tcf) activity, which is closely regulated by the Wnt signaling pathway [111]. This pathway plays a crucial role in maintaining the stem cell niche located at the base of the colonic crypt. Despite being reviewed in this work separately (see Section 4.5), it is noteworthy that it governs apoptosis in this type of cancer.

Under normal conditions, when Wnt signals are absent, β-catenin is bound in a complex with several other proteins, including glycogen synthase kinase 3β (GSK3β), axin/conductin, and adenomatous polyposis coli (APC). This complex leads to the rapid degradation of β-catenin [103]. GSK3β specifically targets β-catenin for destruction through a process known as ubiquitination. However, the presence of Wnt proteins, which are secreted by myofibroblasts around the crypt base, alters this scenario. These proteins bind to Frizzled receptors on the crypt epithelial cells, inhibiting GSK3β activity and thereby prevent the degradation of β-catenin [111,112].

As a result of this inhibition, β-catenin accumulates and moves from the cytosol to the nucleus. There, it binds to Tcf/Lef1 family members, activating a genetic program with significant implications. One key outcome is the up-regulation of cell proliferation and differentiation: when Tcf/β-catenin activity is high (‘on’), cell proliferation is promoted and differentiation is suppressed; when it is low (‘off’), the opposite occurs [113]. c-MYC, a gene activated by Tcf, drives cell proliferation; reducing c-MYC activity leads to cell cycle arrest through the increased activity of the cell cycle inhibitor p21WAF1/CIP1.

Interestingly, the tendency of cells at the crypt base to undergo apoptosis, especially following DNA damage, is not fully explained by current data [111]. However, it is speculated that this phenomenon might also be under the influence of Wnt signaling. This intricate network of molecular signals and pathways underscores the complexity of cellular regulation in colorectal cancer and highlights potential targets for therapeutic intervention.

Nevertheless, to assess apoptosis, concentrations of the key players of the intrinsic and extrinsic apoptotic mechanisms were assessed [80,82,83,84,88,91,94,95].

The intrinsic pathway is triggered by various forms of cellular stress. Its activation is controlled by specific members of the Bcl-2 family. This pathway is regulated primarily at two critical points: firstly, the release of cytochrome c from the mitochondria, and secondly, the activity of caspases, which are enzymes that play a vital role in apoptosis. The initiator caspase for the intrinsic pathway is caspase 9 [111].

On the other hand, the extrinsic pathway plays a crucial role in the immune system. It is activated through the binding of certain ligands to specific receptors on the cell surface, known as “death receptors.” An example of such a receptor is the Fas receptor (FasR). The initiator caspase for the extrinsic pathway is caspase 8 [111].

Chlorogenic acid is observed to promote both the intrinsic and the extrinsic pathway, depending on the types of cells cultured and the dominant mechanism (Table 3). It is observed that the mitochondrion is generally damaged and a promoter for apoptosis, and that the phytochemicals can also induce enhanced oxidative stress (see Section 4.8), despite generally being regarded as antioxidant agents.

### 4.4. Chlorogenic Acids Impact the Migration and Invasion of Colorectal Cancer Models

The role of proteases, particularly gelatinases like matrix metalloproteinase-2 (MMP-2) and matrix metalloproteinase-9 (MMP-9), is increasingly recognized as critical in the progression and metastasis of CRC, both in animal models and human patients. These enzymes are specialized in degrading components of the extracellular matrix, a key step in cancer progression [114].

Gelatinases, such as MMP-2 and MMP-9, facilitate the invasion and spread of cancer cells by breaking down the extracellular matrix and basal membrane barriers. This degradation is a prerequisite for tumor cells to invade neighboring tissues and eventually spread to distant sites, a process known as metastasis.

The downregulation of MMPs by chlorogenic acids [98] suggests the potential of the chemicals as chemotherapy agents, as they would slow down the progression of CRC (Table 4).

The vascular endothelial growth factor (VEGF) family, comprising VEGF-A, VEGF-B, VEGF-C, VEGF-D, and placental growth factor (PlGF), are integral to both physiological and pathological processes involving blood vessel formation. These factors are crucial during embryonic development for the formation of the vascular system [115]. However, they also play a significant role in pathological angiogenesis and lymphangiogenesis, which are processes that enable tumor growth.

The blockade of VEGF-related pathways by chlorogenic acids [90,101] has shown promise and could become an integral part of the therapeutic arsenal against CRC. It could be a strategy that targets the tumor’s lifeline—its blood supply—making it a potent and targeted approach.

### 4.5. Chlorogenic Acids Modulate the Wnt/β-Catenin Pathway in Colorectal Cancer Models

The Wnt/β-catenin pathway was described in detail previously, due to its involvement in apoptosis (see Section 4.3). The chlorogenic acid-induced decrease in β-catenin, Tcf-4, and E-cadherin (Table 5) is in accordance with a beneficial, anti-tumor therapy [81,103].

### 4.6. Chlorogenic Acids Modulate the MAPK/NFκB Pathway in Colorectal Cancer Models

The nuclear factor-kappa B (NF-κB) signaling pathway is a crucial regulator of inflammation and has been implicated in the process of carcinogenesis, particularly in CRC [116]. This pathway involves a family of NF-κB proteins, consisting of five types of hetero- or homodimers. In their inactive state, NF-κB dimers are bound to specific inhibitors, known as inhibitors of NF-κB (IκB), which include inhibitory κB kinases (IKK) or their inactive precursors (p105 and p100) [117].

The activation of NF-κB occurs through two primary pathways: the canonical and the non-canonical pathways. The canonical, or classical, pathway is triggered by extrinsic stimuli like cytokines (IL-1, TNF), T-cell receptors (TCR), or B-cell receptors (BCR). These stimuli activate the IKK complex, leading to the phosphorylation of p105, which then releases the NF-κB dimers [118]. These dimers travel to the nucleus to activate gene expression. The canonical pathway is typically associated with the body’s acute phase response to stimuli.

In the context of colon cancer, the role of NF-κB is particularly significant. Research indicates that IKKB-induced NF-κB activation in intestinal epithelial cells and the resulting inflammation are vital in tumor formation. This finding underlines the importance of the NF-κB pathway in the development and progression of CRC, presenting it as a potential target for therapeutic intervention. The link between inflammation and cancer progression through the NF-κB pathway should be considered when reading Section 4.9.

Mitogen-activated protein kinases (MAPKs) are vital components in cellular signaling, expressed in all cell types and involved in regulating a myriad of physiological processes including cell growth, metabolism, differentiation, and cell death. In mammals, six distinct groups of MAPKs have been characterized: the extracellular signal-regulated kinases (ERK) 1/2, ERK 3/4, ERK5, ERK 7/8, the Jun N-terminal kinases (JNK) 1/2/3, and the p38 MAPKs (p38α/β/γ/δ) [119].

The MAPK signaling pathway is organized as a cascade where the activation of upstream kinases by receptors triggers the sequential activation of a MAPK module, typically composed of MAPKKK (MAPK kinase kinase), MAPKK (MAPK kinase), and MAPK. Crucial to this process are the specific interactions between MAPKs and their substrates, which ensure the accurate transmission of signaling inputs and outputs.

In the context of CRC, the MAPK pathway has been identified as a key factor influencing therapy and chemoresistance. The potential of targeting MAPK signaling for therapeutic interventions through chlorogenic acids [80,83,90,98,100,107] could be explored based on the in vitro results presented below (Table 6).

### 4.7. Chlorogenic Acids Modulate the PI3K/AKT Pathway in Colorectal Cancer Models

The phosphatidylinositide-3-kinase (PI3K)/protein kinase B (Akt) pathway plays a crucial role in the development and progression of various solid cancers, including CRC. Its significance lies in its central function in cell proliferation and survival, which may contribute to more aggressive tumor behavior and enhanced metastatic potential in colon cancer [120]. Analyzing the activation of the PI3K/Akt pathway can offer valuable insights into potential biomarkers for poor prognosis with more aggressive and advanced stage of the disease.

Moreover, there is growing interest in targeting the PI3K/Akt pathway for cancer therapy (Table 7). Recent studies have shown promising results using PI3K/mammalian target of rapamycin (mTOR) inhibitors in treating cancers such as pancreatic cancer, melanomas, and B-cell malignancies [121,122,123]. Similarly, chlorogenic acid downregulates p-Akt [98,100,107], and could therefore be tested for its anti-tumorigenic activity in CRC.

### 4.8. Chlorogenic Acids Induce Oxidative Stress in Colorectal Cancer Models

Colorectal cancer (CRC) cells often exhibit elevated levels of reactive oxygen species (ROS), which are known to promote cell proliferation. In the more advanced stages of CRC, these cells undergo redox adaptation, a process that aids their survival and contributes to drug resistance [124].

The high levels of oxidative stress in tumor cells present a unique therapeutic opportunity. Redox modulation strategies aim to exploit this vulnerability by selectively targeting cancer cells while sparing normal cells. This approach is based on the principle that while both normal and cancer cells experience oxidative stress, cancer cells are closer to the toxic threshold. Thus, by further enhancing ROS levels or by inhibiting ROS elimination in these cells, one can selectively push them over this threshold, leading to their death [124].

Therefore, despite the counteractive idea that chlorogenic acids represent molecules with antioxidant function, it might be beneficial that they increase the levels of ROS in CRC cells (Table 8) [81,82,84,87,89,90,91].

### 4.9. Chlorogenic Acids Modulates the Inflammation in Colorectal Cancer Models

Colorectal tumors are characterized by significant inflammatory infiltration, involving a diverse array of immune cells [125]. This infiltration is not just a byproduct of the tumor environment, playing an active role in the progression of the disease. The various types of immune cells that infiltrate colorectal tumors include macrophages, neutrophils, lymphocytes, and other cell types. Each of these cells can contribute to the tumor microenvironment in different ways.

A key aspect of these infiltrating immune cells is their role as sources of pro-tumorigenic inflammatory cytokines and chemokines. In the context of colorectal cancer, these molecules can create an environment that supports tumor growth and development, through promotion of cell proliferation, angiogenesis, and suppression of the anti-tumor immune response [125].

In all studies that took the inflammatory response into account (Table 9), chlorogenic acids decreased the levels of chemokines and cytokines [88,90,93,100,101,106].

## 5. Conclusions

Chlorogenic acids, as the main constituents of coffee—the most popular drink in the world—should be further assessed for their anti-tumorigenic effect in CRC, based on the presented studies. Even though data from clinical trials are scarce and inconclusive, their potential benefits have been observed in numerous in vitro studies. Noteworthily, the anti-proliferative and pro-apoptotic activity is well documented in various cell culture models representative for the pathology. Moreover, molecular data support the observations regarding the decreased viability of CRC model cells upon treatment with chlorogenic acids.

## Figures and Tables

**Table 1:** Impact of chlorogenic acids on cell viability in colorectal cancer models.

Trend (↑ ↓)	Study Model	Reference, Year
↓	HT-29—cell culture	[80], 2023
↓	HT-29—cell culture	[81], 2022
↓	SW480—cell culture	[81], 2022
↓	HT-29—cell culture	[82], 2023
↓	SW480—cell culture	[82], 2023
↓	HCT116	[83], 2022
↓	HT-29—cell culture	[84], 2021
↓	HT-29—cell culture	[85], 2017
↓	SW480—cell culture	[86], 2021
↓	SW620—cell culture	[86], 2021
↓	HCT116—cell culture	[89], 2017
↓	HT-29—cell culture	[89], 2017
↓	LPS-induced SW480—cell culture	[90], 2023
↓	Caco-2—cell culture	[91], 2022
↓	CT26—cell culture	[91], 2022
↓	HT-29—cell culture	[92], 2021
↓	HT-29—cell culture	[93], 2023
↓	HT-29—cell culture	[95], 2021
↓	RKO—cell culture	[95], 2021
↓	Caco-2—cell culture	[96], 2023
↓	HT-29—cell culture	[97], 2020
↓	SW480—cell culture	[98], 2022
↓	HT-29—cell culture	[98], 2022
↓	SW480—cell culture	[99], 2020
↓	HT-29—cell culture	[99], 2020
↓	Sprague Dawley AOM-induced rats	[100], 2016
↓	HCT116—cell culture	[101], 2024
↓	DLD-1—cell culture	[102], 2007
↓	HCT-116—cell culture	[103], 2014
↓	HT-29—cell culture	[104], 2015
↓	HT-29—cell culture	[105], 2010
↓	HT-29—cell culture	[106], 2011
↓	RKO—cell culture	[106], 2011
↓	HT-29—cell culture	[107], 2011

Note: ↑ = up-regulation; ↓ = down-regulation.

**Table 2:** Impact of chlorogenic acids on the cell cycle in colorectal cancer models.

Trend (↑ ↓)/Target	Study Model	Reference, Year
↑ p21 ↑ p53	HT-29—cell culture	[80], 2023
↓ cyclin-D1	HT-29—cell culture	[81], 2022
↓ cyclin-D1	SW480—cell culture	[81], 2022
↑ sub-G1 cell population	HT-29—cell culture	[82], 2023
↑ sub-G1 cell population	SW480—cell culture	[82], 2023
↑ p53 ↑ p21 ↑ p18 ↑ CDKI ↓ cyclin-D1 ↑ G1 cell population ↓ S cell population ↓ G2 cell population	HCT116—cell culture	[83], 2022
↑ S cell population	HT-29—cell culture	[84], 2021
↑ G1 cell population	HT-29—cells cultured in a 3D model	[87], 2023
↓ Ki-67	male Swiss mice submitted to a 1,2-dimethylhydrazine/deoxycholic acid (DMH/DCA)-induced colon carcinogenesis	[88], 2022
↑ S cell population	HCT116—cell culture	[89], 2017
↑ S cell population	HT-29—cell culture	[89], 2017
↑ p53 ↑ Keap1	Caco-2—cell culture	[91], 2022
↑ sub-G1 cell population	HT-29—cell culture	[95], 2021
↑ sub-G1 cell population	RKO—cell culture	[95], 2021
↑ sub-G1 cell population	HT-29—cell culture	[104], 2015
↑ sub-G1 cell population ↑ p27 ↓ cyclin-D1 ↓ p53	HT-29—cell culture	[107], 2011

Note: ↑ = up-regulation; ↓ = down-regulation.

**Table 3:** Impact of chlorogenic acids on apoptosis in colorectal cancer models.

Trend (↑ ↓)/Target	Study Model	Reference, Year
↑ Bcl-2 ↑ caspase-3 ↑ caspase-9	HT-29—cell culture	[80], 2023
↑ DNA fragmentation	HT-29—cell culture	[82], 2023
↑ DNA fragmentation	SW480—cell culture	[82], 2023
↑ caspase-3 ↑ P38 ↓ ratio of Bcl-2/Bax ↑ DNA fragmentation	HCT116—cell culture	[83], 2022
↓ Bcl-2 ↑ Bax ↑ DNA fragmentation	HT-29—cell culture	[84], 2021
↑ caspase-3	Male Swiss mice submitted to a 1,2-dimethylhydrazine/deoxycholic acid (DMH/DCA)-induced colon carcinogenesis	[88], 2022
↑ Ca^2+^ levels	Caco-2—cell culture	[91], 2022
↑ Ca^2+^ levels	CT26—cell culture	[91], 2022
↓ histone-deacetylase (HDAC)	HT-29—cell culture	[94], 2022
↑ DNA fragmentation	HT-29—cell culture	[95], 2021
↑ DNA fragmentation	RKO—cell culture	[95], 2021
↑ DNA fragmentation	HT-29—cell culture	[104], 2015
↑ caspase-8 ↑ caspase-3	RKO—cell culture	[106], 2011
↓ ratio of Bcl-2/Bax ↑ caspase-8 ↑ caspase-3	HT-29—cell culture	[106], 2011
↑ caspase-3 ↑ PARP ↑ Cytochrome-C ↓ ratio of Bcl-2/Bax	HT-29—cell culture	[107], 2011

Note: ↑ = up-regulation; ↓ = down-regulation.

**Table 4:** Impact of chlorogenic acids on the migration and invasion of colorectal cancer models.

Trend (↑ ↓)/Target	Study Model	Reference, Year
↓	HT-29—cell culture	[81], 2022
↓	SW480—cell culture	[81], 2022
↓	HT-29—cell culture	[84], 2021
↓	SW480—cell culture	[86], 2021
↓	SW620—cell culture	[86], 2021
↓ VEGFC	LPS-induced SW480—cell culture	[90], 2023
↓	HT-29—cell culture	[93], 2023
↓ MMP-2 ↑ MMP-9	SW480—cell culture	[98], 2022
↓ MMP-2 ↓ MMP-9	HT-29—cell culture	[98], 2022
↓ VEGFA	HCT116—cell culture	[101], 2024

Note: ↑ = up-regulation; ↓ = down-regulation.

**Table 5:** Impact of chlorogenic acids on the Wnt/β-catenin pathway in colorectal cancer models.

Trend (↑ ↓)/Target	Study Model	Reference, Year
↓ β-catenin ↓ E-cadherin	HT-29—cell culture	[81], 2022
↓ β-catenin ↓ E-cadherin	SW480—cell culture	[81], 2022
↓ Tcf-4	HCT-116—cell culture	[103], 2014

Note: ↑ = up-regulation; ↓ = down-regulation.

**Table 6:** Impact of chlorogenic acids on the MAPK/NFκB pathway in colorectal cancer models.

Trend (↑ ↓)/Target	Study Model	Reference, Year
↑ NF-κB	HT-29—cell culture	[80], 2023
↓ AP-1 ↓ NFκB ↓ JNK ↓ ERK ↓ MAPK	HCT116—cell culture	[83], 2022
↓ NFκB ↓ Toll-like receptor 4 (TLR4)	LPS-induced SW480—cell culture	[90], 2023
↑ p-ERK	SW480—cell culture	[98], 2022
↑ p-ERK	HT-29—cell culture	[98], 2022
↓ NFκB	Sprague Dawley AOM-induced rats	[100], 2016
↓ ERK ↓ NFκB	HT-29—cell culture	[107], 2011

Note: ↑ = up-regulation; ↓ = down-regulation.

**Table 7:** Impact of chlorogenic acids on PI3K/AKT pathway in colorectal cancer models.

Trend (↑ ↓)/Target	Study Model	Reference, Year
↓ p-AKT	SW480—cell culture	[98], 2022
↓ p-AKT	HT-29—cell culture	[98], 2022
↓ p-AKT ↓ mTOR	Sprague Dawley AOM-induced rats	[100], 2016
↓ p-AKT	HT-29—cell culture	[107], 2011

Note: ↑ = up-regulation; ↓ = down-regulation.

**Table 8:** Impact of chlorogenic acids on oxidative stress in colorectal cancer models.

Trend (↑ ↓)/Target	Study Model	Reference, Year
↑	HT-29—cell culture	[81], 2022
↑	SW480—cell culture	[81], 2022
↑	HT-29—cell culture	[82], 2023
↑	SW480—cell culture	[82], 2023
↑	HT-29—cell culture	[84], 2021
↑	HT-29 cells cultured in a 3D model	[87], 2023
↑	HCT116—cell culture	[89], 2017
↑	HT-29—cell culture	[89], 2017
↓	LPS-induced SW480—cell culture	[90], 2023
↑ ↑ GSH ↑ Nrf-2 ↑ HO-1	Caco-2—cell culture	[91], 2022
↑ ↑ GSH ↑ Nrf-2 ↑ HO-1	CT26—cell culture	[91], 2022

Note: ↑ = up-regulation; ↓ = down-regulation.

**Table 9:** Impact of chlorogenic acids on inflammation in colorectal cancer models.

Trend (↑ ↓)/Target	Study Model	Reference, Year
↓ IL-6 ↓ IL-17 ↓ TNF-a	male Swiss 1,2-dimethylhydrazine/deoxycholic acid (DMH/DCA)-induced mice	[88], 2022
↓ COX-2 ↓ TNF-α ↓ IL-1β ↓ IL-6	LPS-induced SW480—cell culture	[100], 2016
↓ COX-2 ↓ TNF-α	HT-29—cell culture	[93], 2023
↓ NO synthetase ↓ COX-2	Sprague Dawley AOM-induced rats	[100], 2016
↓ TNF-α ↓ HIF1-α	HCT116—cell culture	[101], 2024
↓ NO ↓ iNOS ↓ prostaglandin E(2) ↓ COX-2 ↓ TNF-α ↓ p50 ↓ p65	LPS-induced RAW 264.7 macrophage inflammation	[106], 2011

Note: ↑ = up-regulation; ↓ = down-regulation.

## Data Availability

The Supplementary Information contains the articles that pertain to the systematic literature review presented in Section 4.

## Supplementary Materials

The following supporting information can be downloaded at: https://www.mdpi.com/article/10.3390/cimb46070405/s1.
