# Supplementary material for: A Comprehensive View on the Impact of Chlorogenic Acids on Colorectal Cancer
Source: Curr Issues Mol Biol. 2024 Jul 2;46(7):6783–804. doi: 10.3390/cimb46070405 (PMC11276415; doi:10.3390/cimb46070405)
Supplement: Supplementary file 1 [file cimb-46-00405-s001.zip › cimb-3021443-supplementary.pdf]

Supplementary Materials Bibliography (1)

1: Deshmukh R, Prajapati M, Harwansh RK. Management of Colorectal Cancer Using Nanocarriers-based Drug Delivery for Herbal Bioactives: Current and Emerging Approaches. Curr Pharm Biotechnol. 2024;25(5):599-622. doi: 10.2174/0113892010242028231002075512. PMID: 38807329.

2: Gutierrez-Silerio GY, Garcia-Solis P, Yahia EM, Núñez-Ríos JD, Vázquez-Cuevas F, Rodriguez-Salinas PA, Mendoza-Zuñiga R, Kuri-García A. Cytotoxic and Antitumoral Effects of Methanolic Extracts of Avocado Fruit Mesocarp in Colorectal Cancer Cell Line HT29. J Med Food. 2024 Mar;27(3):211-221. doi: 10.1089/jmf.2023.0112. Epub 2024 Feb 26. PMID: 38407926.

3: Chiavaroli A, Masciulli F, Ingallina C, Mannina L, Loreta Libero M, Di Simone SC, Acquaviva A, Nilofar, Recinella L, Leone S, Brunetti L, Carradori S, Cantò L, Orlando G, Zengin G, Ibrahim Uba A, Cakilcioğlu U, Mukemre M, Elkiran O, Di Vito M, Menghini L, Ferrante C. Comprehensive metabolite and biological profile of "Sulmona Red Garlic" ecotype's aerial bulbils. Food Res Int. 2024 Jan;175:113654. doi: 10.1016/j.foodres.2023.113654. Epub 2023 Nov 2. PMID: 38129017.

4: Ranjbary AG, Bagherzadeh A, Sabbaghi SS, Faghihi A, Karimi DN, Naji S, Kardani M. Chlorogenic acid induces apoptosis and cell-cycle arrest in colorectal cancer cells. Mol Biol Rep. 2023 Dec;50(12):9845-9857. doi: 10.1007/s11033-023-08854-y. Epub 2023 Oct 17. PMID: 37847443.

5: Vélez MD, Pedroza-Díaz J, Santa-González GA. Data on the cytotoxicity of chlorogenic acid in 3D cultures of HT-29 cells. Data Brief. 2023 Aug 28;50:109527. doi: 10.1016/j.dib.2023.109527. PMID: 37691736; PMCID: PMC10482754.

6: Sowa I, Mołdoch J, Paduch R, Strzemski M, Szkutnik J, Tyszczuk-Rotko K, Dresler S, Szczepanek D, Wójciak M. Polyphenolic Composition of <i>Carlina acaulis</i> L. Extract and Cytotoxic Potential against Colorectal Adenocarcinoma and Cervical Cancer Cells. Molecules. 2023 Aug 20;28(16):6148. doi: 10.3390/molecules28166148. PMID: 37630400; PMCID: PMC10458490.

7: Panyathep A, Punturee K, Chewonarin T. Inhibitory Effects of Chlorogenic Acid Containing Green Coffee Bean Extract on Lipopolysaccharide-Induced Inflammatory Responses and Progression of Colon Cancer Cell Line. Foods. 2023 Jul 9;12(14):2648. doi: 10.3390/foods12142648. PMID: 37509740; PMCID: PMC10378980.

8: Keskin C, Ölçekçi A, Baran A, Baran MF, Eftekhari A, Omarova S, Khalilov R, Aliyev E, Sufianov A, Beilerli A, Gareev I. Green synthesis of silver nanoparticles mediated <i>Diospyros kaki</i> L. (Persimmon): determination of chemical composition and evaluation of their antimicrobials and anticancer activities. Front Chem. 2023 May 30;11:1187808. doi: 10.3389/fchem.2023.1187808. PMID: 37324556; PMCID: PMC10265676.

9: Vélez-Vargas LC, Santa-González GA, Uribe D, Henao-Castañeda IC, Pedroza-Díaz J. In Vitro and In Silico Study on the Impact of Chlorogenic Acid in Colorectal Cancer Cells: Proliferation, Apoptosis, and Interaction with β-Catenin and LRP6. Pharmaceuticals (Basel). 2023 Feb 13;16(2):276. doi: 10.3390/ph16020276. PMID: 37259421; PMCID: PMC9960681.

10: Villada Ramos JA, Aguillón Osma J, Restrepo Cortes B, Loango Chamarro N, Maldonado Celis ME. Identification of potential bioactive compounds of <i>Passiflora edulis</i> leaf extract against colon adenocarcinoma cells. Biochem Biophys Rep. 2023 Mar 28;34:101453. doi: 10.1016/j.bbrep.2023.101453. PMID: 37009402; PMCID: PMC10063390.

11: Yildirim BA, Gedikli S, Kordali S, Kucukaydin S. Apoptotic and antiproliferative effects of Inula viscosa L. water extract in the expression of microRnas on HCT 116 cell line: an in vitro study. Int J Environ Health Res. 2024 Feb;34(2):1076-1087. doi: 10.1080/09603123.2023.2197641. Epub 2023 Mar 30. PMID: 36998228.

12: Villota H, Santa-González GA, Uribe D, Henao IC, Arroyave-Ospina JC, Barrera-Causil CJ, Pedroza-Díaz J. Modulatory Effect of Chlorogenic Acid and Coffee Extracts on Wnt/β-Catenin Pathway in Colorectal Cancer Cells. Nutrients. 2022 Nov 18;14(22):4880. doi: 10.3390/nu14224880. PMID: 36432565; PMCID: PMC9693551.

13: Augustynowicz D, Lemieszek MK, Strawa JW, Wiater A, Tomczyk M. Anticancer potential of acetone extracts from selected <i>Potentilla</i> species against human colorectal cancer cells. Front Pharmacol. 2022 Sep 29;13:1027315. doi: 10.3389/fphar.2022.1027315. PMID: 36249795; PMCID: PMC9556846.

14: Nieto-Figueroa KH, Gaytán-Martínez M, Loarca-Piña MGF, Campos-Vega R. Effect of drying method on the production of in vitro short-chain fatty acids and histone deacetylase mediation of cocoa pod husk. J Food Sci. 2022 Oct;87(10):4476-4490. doi: 10.1111/1750-3841.16309. Epub 2022 Sep 14. PMID: 36102033.

15: Owczarek K, Sosnowska D, Kajszczak D, Lewandowska U. Evaluation of phenolic composition, antioxidant and cytotoxic activity of aronia melanocarpa leaf extracts. J Physiol Pharmacol. 2022 Apr;73(2). doi: 10.26402/jpp.2022.2.06. Epub 2022 Aug 18. PMID: 35988931.

16: Yang Q, Zhang X, Qin H, Luo F, Ren J. Phenolic Acid Profiling of <i>Lactarius hatsudake</i> Extracts, Anti-Cancer Function and Its Molecular Mechanisms. Foods. 2022 Jun 22;11(13):1839. doi: 10.3390/foods11131839. PMID: 35804655; PMCID: PMC9266154.

17: Bartolomeu AR, Romualdo GR, Lisón CG, Besharat ZM, Corrales JAM, Chaves MÁG, Barbisan LF. Caffeine and Chlorogenic Acid Combination Attenuate Early-Stage Chemically Induced Colon Carcinogenesis in Mice: Involvement of oncomiR miR-21a-5p. Int J Mol Sci. 2022 Jun 4;23(11):6292. doi: 10.3390/ijms23116292. PMID: 35682971; PMCID: PMC9181067.

18: Chojnacka K, Owczarek K, Caban M, Sosnowska D, Kajszczak D, Lewandowska U. Chemoprotective effects of Japanese quince (Chaenomeles japonica L.) phenol leaf extract on colon cancer cells through the modulation of extracellular signal-regulated kinases/AKT signaling pathway. J Physiol Pharmacol. 2022 Feb;73(1). doi: 10.26402/jpp.2022.1.04. Epub 2022 May 27. PMID: 35639036.

19: Batjargal A, Solek P, Kukula-Koch W, Urjin B, Koch W, Koman D, Dudzinska E. Gurgem-7 toxicity assessment: Regulation of cell survival or death by traditional Mongolian prescription. Ecotoxicol Environ Saf. 2022 Jul 1;239:113660. doi: 10.1016/j.ecoenv.2022.113660. Epub 2022 May 20. PMID: 35605329.

20: Selvaraj J, Vishnupriya V, Sardar H, Balakrishna JP, Rex J, Mohan SK, Vijayalakshmi P, Ponnulakshmi R. Molecular docking analysis of beta-catenin with compounds derived from Lycopersicon esculentum. Bioinformation. 2020 Nov 30;16(11):801-806. doi: 10.6026/97320630016801. PMID: 34803252; PMCID: PMC8573472.

21: Ayan İÇ, Çetinkaya S, Dursun HG, Güneş CE, Şirin S. Anticancer Effect and Phytochemical Profile of the Extract from Achillea ketenoglui against Human Colorectal Cancer Cell Lines. Anticancer Agents Med Chem. 2022;22(9):1769-1779. doi: 10.2174/1871520621666210908110422. PMID: 34503424.

22: Villota H, Moreno-Ceballos M, Santa-González GA, Uribe D, Castañeda ICH, Preciado LM, Pedroza-Díaz J. Biological Impact of Phenolic Compounds from Coffee on Colorectal Cancer. Pharmaceuticals (Basel). 2021 Aug 3;14(8):761. doi:

10.3390/ph14080761. PMID: 34451858; PMCID: PMC8401378.

23: Mondal A, Banerjee S, Bose S, Das PP, Sandberg EN, Atanasov AG, Bishayee A. Cancer Preventive and Therapeutic Potential of Banana and Its Bioactive Constituents: A Systematic, Comprehensive, and Mechanistic Review. Front Oncol. 2021 Jul 7;11:697143. doi: 10.3389/fonc.2021.697143. PMID: 34307163; PMCID: PMC8294041.

24: Çetinkaya S, Çınar Ayan İ, Süntar İ, Dursun HG. The Phytochemical Profile and Biological Activity of <i>Liquidambar orientalis</i> Mill. var. <i>orientalis</i> via NF-κB and Apoptotic Pathways in Human Colorectal Cancer. Nutr Cancer. 2022;74(4):1457-1473. doi: 10.1080/01635581.2021.1952455. Epub 2021 Jul 22. PMID: 34291706.

25: Albogami S, Hassan AM. Assessment of the Efficacy of Olive Leaf (<i>Olea europaea</i> L.) Extracts in the Treatment of Colorectal Cancer and Prostate Cancer Using In Vitro Cell Models. Molecules. 2021 Jul 3;26(13):4069. doi: 10.3390/molecules26134069. PMID: 34279409; PMCID: PMC8272070.

26: Marzo F, Milagro FI, Barrenetxe J, Díaz MT, Martínez JA. Azoxymethane-Induced Colorectal Cancer Mice Treated with a Polyphenol-Rich Apple Extract Show Less Neoplastic Lesions and Signs of Cachexia. Foods. 2021 Apr 15;10(4):863. doi: 10.3390/foods10040863. PMID: 33921048; PMCID: PMC8071383.

27: Villarini M, Acito M, di Vito R, Vannini S, Dominici L, Fatigoni C, Pagiotti R, Moretti M. Pro-Apoptotic Activity of Artichoke Leaf Extracts in Human HT-29 and RKO Colon Cancer Cells. Int J Environ Res Public Health. 2021 Apr 15;18(8):4166. doi: 10.3390/ijerph18084166. PMID: 33920761; PMCID: PMC8071198.

28: Ayouaz S , Oliveira-Alves SC , Serra AT , Lefsih K , Samah M , Bento da Silva A , Madani K , Bronze MR . LC-DAD-ESI-MS/MS analysis and cytotoxic and antiproliferative effects of chlorogenic acid derivative rich extract from Nerium oleander L. pink flowers. Food Funct. 2021 Apr 21;12(8):3624-3634. doi: 10.1039/d0fo02640a. Epub 2021 Mar 31. PMID: 33900304.

29: Castaldo L, Izzo L, Narváez A, Rodríguez-Carrasco Y, Grosso M, Ritieni A. Colon Bioaccessibility under In Vitro Gastrointestinal Digestion of Different Coffee Brews Chemically Profiled through UHPLC-Q-Orbitrap HRMS. Foods. 2021 Jan 17;10(1):179. doi: 10.3390/foods10010179. PMID: 33477307; PMCID: PMC7829986.

30: Yapıcı İ, Altay A, Öztürk Sarıkaya B, Korkmaz M, Atila A, Gülçin İ, Köksal E. In vitro Antioxidant and Cytotoxic Activities of Extracts of Endemic

Tanacetum erzincanense Together with Phenolic Content by LC-ESI-QTOF-MS. Chem Biodivers. 2021 Mar;18(3):e2000812. doi: 10.1002/cbdv.202000812. Epub 2021 Feb 8. PMID: 33464702.

31: Caicedo-Lopez LH, Cuellar-Nuñez ML, Luzardo-Ocampo I, Campos-Vega R, Lóarca-
Piña G. Colonic metabolites from digested <i>Moringa oleifera</i> leaves induced HT-29 cell death via apoptosis, necrosis, and autophagy. Int J Food Sci Nutr. 2021 Jun;72(4):485-498. doi: 10.1080/09637486.2020.1849039. Epub 2020 Dec 10. PMID: 33302731.

32: Chojnacka K, Sosnowska D, Polka D, Owczarek K, Gorlach-Lira K, Oliveira de Verasa B, Lewandowska U. Comparison of phenolic compounds, antioxidant and cytotoxic activity of extracts prepared from Japanese quince (Chaenomeles japonica L.) leaves. J Physiol Pharmacol. 2020 Apr;71(2). doi: 10.26402/jpp.2020.2.05. Epub 2020 Jul 2. PMID: 32633239.

33: Ayouaz S , Oliveira-Alves SC , Lefsih K , Serra AT , Bento da Silva A , Samah M , Karczewski J , Madani K , Bronze MR . Phenolic compounds from Nerium oleander leaves: microwave assisted extraction, characterization, antiproliferative and cytotoxic activities. Food Funct. 2020 Jul 1;11(7):6319-6331. doi: 10.1039/d0fo01180k. Epub 2020 Jul 1. PMID: 32608462.

34: Santana-Gálvez J, Villela-Castrejón J, Serna-Saldívar SO, Cisneros-Zevallos L, Jacobo-Velázquez DA. Synergistic Combinations of Curcumin, Sulforaphane, and Dihydrocaffeic Acid against Human Colon Cancer Cells. Int J Mol Sci. 2020 Apr 28;21(9):3108. doi: 10.3390/ijms21093108. PMID: 32354075; PMCID: PMC7246525.

35: Taha KF, Khalil M, Abubakr MS, Shawky E. Identifying cancer-related molecular targets of Nandina domestica Thunb. by network pharmacology-based analysis in combination with chemical profiling and molecular docking studies. J Ethnopharmacol. 2020 Mar 1;249:112413. doi: 10.1016/j.jep.2019.112413. Epub 2019 Nov 21. PMID: 31760157.

36: Popović BM, Blagojević B, Ždero Pavlović R, Mićić N, Bijelić S, Bogdanović B, Mišan A, Duarte CMM, Serra AT. Comparison between polyphenol profile and bioactive response in blackthorn (Prunus spinosa L.) genotypes from north Serbia-from raw data to PCA analysis. Food Chem. 2020 Jan 1;302:125373. doi: 10.1016/j.foodchem.2019.125373. Epub 2019 Aug 14. PMID: 31442706.

37: Romualdo GR, Rocha AB, Vinken M, Cogliati B, Moreno FS, Chaves MAG, Barbisan LF. Drinking for protection? Epidemiological and experimental evidence on the

beneficial effects of coffee or major coffee compounds against gastrointestinal and liver carcinogenesis. Food Res Int. 2019 Sep;123:567-589. doi: 10.1016/j.foodres.2019.05.029. Epub 2019 May 22. PMID: 31285007.

38: Bułdak RJ, Hejmo T, Osowski M, Bułdak Ł, Kukla M, Polaniak R, Birkner E. The Impact of Coffee and Its Selected Bioactive Compounds on the Development and Progression of Colorectal Cancer In Vivo and In Vitro. Molecules. 2018 Dec 13;23(12):3309. doi: 10.3390/molecules23123309. PMID: 30551667; PMCID: PMC6321559.

39: Dos Reis Luz L, Porto DD, Castro CB, Silva MFS, de Godoy Alves Filho E, Canuto KM, de Brito ES, Becker H, do Ó Pessoa C, Zocolo GJ. Metabolomic profile of Schinopsis brasiliensis via UPLC-QTOF-MS for identification of biomarkers and evaluation of its cytotoxic potential. J Chromatogr B Analyt Technol Biomed Life Sci. 2018 Nov 1;1099:97-109. doi: 10.1016/j.jchromb.2018.09.019. Epub 2018 Sep 18. PMID: 30265941.

40: Agudelo CD, Luzardo-Ocampo I, Campos-Vega R, Loarca-Piña G, Maldonado-Celis ME. Bioaccessibility during In Vitro Digestion and Antiproliferative Effect of Bioactive Compounds from Andean Berry (Vaccinium meridionale Swartz) Juice. J Agric Food Chem. 2018 Jul 18;66(28):7358-7366. doi: 10.1021/acs.jafc.8b01604. Epub 2018 Jul 6. PMID: 29913068.

41: Zhang S, Yang C, Idehen E, Shi L, Lv L, Sang S. Novel Theaflavin-Type Chlorogenic Acid Derivatives Identified in Black Tea. J Agric Food Chem. 2018 Apr 4;66(13):3402-3407. doi: 10.1021/acs.jafc.7b06044. Epub 2018 Mar 21. PMID: 29534564.

42: Cuellar-Nuñez ML, Luzardo-Ocampo I, Campos-Vega R, Gallegos-Corona MA, González de Mejía E, Loarca-Piña G. Physicochemical and nutraceutical properties of moringa (Moringa oleifera) leaves and their effects in an in vivo AOM/DSS-induced colorectal carcinogenesis model. Food Res Int. 2018 Mar;105:159-168. doi: 10.1016/j.foodres.2017.11.004. Epub 2017 Nov 6. PMID: 29433203.

43: Vancsik T, Kovago C, Kiss E, Papp E, Forika G, Benyo Z, Meggyeshazi N, Krenacs T. Modulated electro-hyperthermia induced loco-regional and systemic tumor destruction in colorectal cancer allografts. J Cancer. 2018 Jan 1;9(1):41-53. doi: 10.7150/jca.21520. PMID: 29290768; PMCID: PMC5743710.

44: Nam SH, Ko JA, Jun W, Wee YJ, Walsh MK, Yang KY, Choi JH, Eun JB, Choi J, Kim YM, Han S, Nguyen TTH, Kim D. Enzymatic synthesis of chlorogenic acid glucoside using dextransucrase and its physical and functional properties.

Enzyme Microb Technol. 2017 Dec;107:15-21. doi: 10.1016/j.enzmictec.2017.07.011. Epub 2017 Jul 29. PMID: 28899482.

45: Martinez-Saez N, Hochkogler CM, Somoza V, Del Castillo MD. Biscuits with No Added Sugar Containing Stevia, Coffee Fibre and Fructooligosaccharides Modifies α-Glucosidase Activity and the Release of GLP-1 from HuTu-80 Cells and Serotonin from Caco-2 Cells after In Vitro Digestion. Nutrients. 2017 Jul 4;9(7):694. doi: 10.3390/nu9070694. PMID: 28677657; PMCID: PMC5537809.

46: Ombra MN, d'Acierno A, Nazzaro F, Riccardi R, Spigno P, Zaccardelli M, Pane C, Maione M, Fratianni F. Phenolic Composition and Antioxidant and Antiproliferative Activities of the Extracts of Twelve Common Bean (<i>Phaseolus vulgaris</i> L.) Endemic Ecotypes of Southern Italy before and after Cooking. Oxid Med Cell Longev. 2016;2016:1398298. doi: 10.1155/2016/1398298. Epub 2016 Dec 25. PMID: 28105248; PMCID: PMC5220516.

47: Banerjee N, Kim H, Talcott ST, Turner ND, Byrne DH, Mertens-Talcott SU. Plum polyphenols inhibit colorectal aberrant crypt foci formation in rats: potential role of the miR-143/protein kinase B/mammalian target of rapamycin axis. Nutr Res. 2016 Oct;36(10):1105-1113. doi: 10.1016/j.nutres.2016.06.008. Epub 2016 Jun 14. PMID: 27865352.

48: Hou N, Liu N, Han J, Yan Y, Li J. Chlorogenic acid induces reactive oxygen species generation and inhibits the viability of human colon cancer cells. Anticancer Drugs. 2017 Jan;28(1):59-65. doi: 10.1097/CAD.0000000000000430. PMID: 27603595.

49: Lee J, Kim YS, Lee J, Heo SC, Lee KL, Choi SW, Kim Y. Walnut Phenolic Extract and Its Bioactive Compounds Suppress Colon Cancer Cell Growth by Regulating Colon Cancer Stemness. Nutrients. 2016 Jul 21;8(7):439. doi: 10.3390/nu8070439. PMID: 27455311; PMCID: PMC4963915.

50: Mira A, Shimizu K. In vitro Cytotoxic Activities and Molecular Mechanisms of Angelica shikokiana Extract and its Isolated Compounds. Pharmacogn Mag. 2015 Oct;11(Suppl 4):S564-9. doi: 10.4103/0973-1296.172962. PMID: 27013795; PMCID: PMC4787089.

51: Van Hecke T, Wouters A, Rombouts C, Izzati T, Berardo A, Vossen E, Claeys E, Van Camp J, Raes K, Vanhaecke L, Peeters M, De Vos WH, De Smet S. Reducing Compounds Equivocally Influence Oxidation during Digestion of a High-Fat Beef Product, which Promotes Cytotoxicity in Colorectal Carcinoma Cell Lines. J Agric Food Chem. 2016 Feb 24;64(7):1600-9. doi: 10.1021/acs.jafc.5b05915. Epub 2016

Feb 12. PMID: 26836477.

52: Choi DW, Lim MS, Lee JW, Chun W, Lee SH, Nam YH, Park JM, Choi DH, Kang CD, Lee SJ, Park SC. The Cytotoxicity of Kahweol in HT-29 Human Colorectal Cancer Cells Is Mediated by Apoptosis and Suppression of Heat Shock Protein 70 Expression. Biomol Ther (Seoul). 2015 Mar;23(2):128-33. doi: 10.4062/biomolther.2014.133. Epub 2015 Mar 1. PMID: 25767680; PMCID: PMC4354313.

Supplementary Materials Bibliography (2)

1: Castaldo L, Izzo L, Narváez A, Rodríguez-Carrasco Y, Grosso M, Ritieni A. Colon Bioaccessibility under In Vitro Gastrointestinal Digestion of Different Coffee Brews Chemically Profiled through UHPLC-Q-Orbitrap HRMS. Foods. 2021 Jan 17;10(1):179. doi: 10.3390/foods10010179. PMID: 33477307; PMCID: PMC7829986.

2: Popović BM, Blagojević B, Ždero Pavlović R, Mićić N, Bijelić S, Bogdanović B, Mišan A, Duarte CMM, Serra AT. Comparison between polyphenol profile and bioactive response in blackthorn (Prunus spinosa L.) genotypes from north Serbia-from raw data to PCA analysis. Food Chem. 2020 Jan 1;302:125373. doi: 10.1016/j.foodchem.2019.125373. Epub 2019 Aug 14. PMID: 31442706.

3: Banerjee N, Kim H, Talcott ST, Turner ND, Byrne DH, Mertens-Talcott SU. Plum polyphenols inhibit colorectal aberrant crypt foci formation in rats: potential role of the miR-143/protein kinase B/mammalian target of rapamycin axis. Nutr Res. 2016 Oct;36(10):1105-1113. doi: 10.1016/j.nutres.2016.06.008. Epub 2016 Jun 14. PMID: 27865352.

4: Murad LD, Soares Nda C, Brand C, Monteiro MC, Teodoro AJ. Effects of caffeic and 5-caffeoylquinic acids on cell viability and cellular uptake in human colon adenocarcinoma cells. Nutr Cancer. 2015;67(3):532-42. doi: 10.1080/01635581.2015.1004736. Epub 2015 Mar 24. PMID: 25803129.

Supplementary Materials Bibliography (3)

1: Murad LD, Soares Nda C, Brand C, Monteiro MC, Teodoro AJ. Effects of caffeic and 5-caffeoylquinic acids on cell viability and cellular uptake in human colon adenocarcinoma cells. Nutr Cancer. 2015;67(3):532-42. doi: 10.1080/01635581.2015.1004736. Epub 2015 Mar 24. PMID: 25803129.

2: Gutierrez-Silerio GY, Garcia-Solis P, Yahia EM, Núñez-Ríos JD, Vázquez-Cuevas F, Rodriguez-Salinas PA, Mendoza-Zuñiga R, Kuri-García A. Cytotoxic and Antitumoral Effects of Methanolic Extracts of Avocado Fruit Mesocarp in Colorectal Cancer Cell Line HT29. J Med Food. 2024 Mar;27(3):211-221. doi: 10.1089/jmf.2023.0112. Epub 2024 Feb 26. PMID: 38407926.

3: Chiavaroli A, Masciulli F, Ingallina C, Mannina L, Loreta Libero M, Di Simone SC, Acquaviva A, Nilofar, Recinella L, Leone S, Brunetti L, Carradori S, Cantò L, Orlando G, Zengin G, Ibrahim Uba A, Cakilcioğlu U, Mukemre M, Elkiran O, Di Vito M, Menghini L, Ferrante C. Comprehensive metabolite and biological profile of "Sulmona Red Garlic" ecotype's aerial bulbils. Food Res Int. 2024 Jan;175:113654. doi: 10.1016/j.foodres.2023.113654. Epub 2023 Nov 2. PMID: 38129017.

4: Ranjbary AG, Bagherzadeh A, Sabbaghi SS, Faghihi A, Karimi DN, Naji S, Kardani M. Chlorogenic acid induces apoptosis and cell-cycle arrest in colorectal cancer cells. Mol Biol Rep. 2023 Dec;50(12):9845-9857. doi: 10.1007/s11033-023-08854-y. Epub 2023 Oct 17. PMID: 37847443.

5: Vélez MD, Pedroza-Díaz J, Santa-González GA. Data on the cytotoxicity of chlorogenic acid in 3D cultures of HT-29 cells. Data Brief. 2023 Aug 28;50:109527. doi: 10.1016/j.dib.2023.109527. PMID: 37691736; PMCID: PMC10482754.

6: Sowa I, Mołdoch J, Paduch R, Strzemski M, Szkutnik J, Tyszczuk-Rotko K, Dresler S, Szczepanek D, Wójciak M. Polyphenolic Composition of <i>Carlina acaulis</i> L. Extract and Cytotoxic Potential against Colorectal Adenocarcinoma and Cervical Cancer Cells. Molecules. 2023 Aug 20;28(16):6148. doi: 10.3390/molecules28166148. PMID: 37630400; PMCID: PMC10458490.

7: Panyathep A, Punturee K, Chewonarin T. Inhibitory Effects of Chlorogenic Acid Containing Green Coffee Bean Extract on Lipopolysaccharide-Induced Inflammatory Responses and Progression of Colon Cancer Cell Line. Foods. 2023 Jul 9;12(14):2648. doi: 10.3390/foods12142648. PMID: 37509740; PMCID: PMC10378980.

8: Keskin C, Ölçekçi A, Baran A, Baran MF, Eftekhari A, Omarova S, Khalilov R, Aliyev E, Sufianov A, Beilerli A, Gareev I. Green synthesis of silver nanoparticles mediated <i>Diospyros kaki</i> L. (Persimmon): determination of chemical composition and evaluation of their antimicrobials and anticancer activities. Front Chem. 2023 May 30;11:1187808. doi: 10.3389/fchem.2023.1187808. PMID: 37324556; PMCID: PMC10265676.

9: Vélez-Vargas LC, Santa-González GA, Uribe D, Henao-Castañeda IC, Pedroza-Díaz J. In Vitro and In Silico Study on the Impact of Chlorogenic Acid in Colorectal Cancer Cells: Proliferation, Apoptosis, and Interaction with β-Catenin and LRP6. Pharmaceuticals (Basel). 2023 Feb 13;16(2):276. doi: 10.3390/ph16020276. PMID: 37259421; PMCID: PMC9960681.

10: Villada Ramos JA, Aguillón Osma J, Restrepo Cortes B, Loango Chamarro N, Maldonado Celis ME. Identification of potential bioactive compounds of <i>Passiflora edulis</i> leaf extract against colon adenocarcinoma cells. Biochem Biophys Rep. 2023 Mar 28;34:101453. doi: 10.1016/j.bbrep.2023.101453. PMID: 37009402; PMCID: PMC10063390.

11: Yildirim BA, Gedikli S, Kordali S, Kucukaydin S. Apoptotic and antiproliferative effects of Inula viscosa L. water extract in the expression of microRnas on HCT 116 cell line: an in vitro study. Int J Environ Health Res. 2024 Feb;34(2):1076-1087. doi: 10.1080/09603123.2023.2197641. Epub 2023 Mar 30. PMID: 36998228.

12: Villota H, Santa-González GA, Uribe D, Henao IC, Arroyave-Ospina JC, Barrera-Causil CJ, Pedroza-Díaz J. Modulatory Effect of Chlorogenic Acid and Coffee Extracts on Wnt/β-Catenin Pathway in Colorectal Cancer Cells. Nutrients. 2022 Nov 18;14(22):4880. doi: 10.3390/nu14224880. PMID: 36432565; PMCID: PMC9693551.

13: Augustynowicz D, Lemieszek MK, Strawa JW, Wiater A, Tomczyk M. Anticancer potential of acetone extracts from selected <i>Potentilla</i> species against human colorectal cancer cells. Front Pharmacol. 2022 Sep 29;13:1027315. doi: 10.3389/fphar.2022.1027315. PMID: 36249795; PMCID: PMC9556846.

14: Nieto-Figueroa KH, Gaytán-Martínez M, Loarca-Piña MGF, Campos-Vega R. Effect of drying method on the production of in vitro short-chain fatty acids and histone deacetylase mediation of cocoa pod husk. J Food Sci. 2022 Oct;87(10):4476-4490. doi: 10.1111/1750-3841.16309. Epub 2022 Sep 14. PMID: 36102033.

15: Owczarek K, Sosnowska D, Kajszczak D, Lewandowska U. Evaluation of phenolic composition, antioxidant and cytotoxic activity of aronia melanocarpa leaf extracts. J Physiol Pharmacol. 2022 Apr;73(2). doi: 10.26402/jpp.2022.2.06. Epub 2022 Aug 18. PMID: 35988931.

16: Yang Q, Zhang X, Qin H, Luo F, Ren J. Phenolic Acid Profiling of <i>Lactarius hatsudake</i> Extracts, Anti-Cancer Function and Its Molecular Mechanisms. Foods. 2022 Jun 22;11(13):1839. doi: 10.3390/foods11131839. PMID: 35804655; PMCID: PMC9266154.

17: Bartolomeu AR, Romualdo GR, Lisón CG, Besharat ZM, Corrales JAM, Chaves MÁG, Barbisan LF. Caffeine and Chlorogenic Acid Combination Attenuate Early-Stage Chemically Induced Colon Carcinogenesis in Mice: Involvement of oncomiR miR-21a-5p. Int J Mol Sci. 2022 Jun 4;23(11):6292. doi: 10.3390/ijms23116292. PMID: 35682971; PMCID: PMC9181067.

18: Chojnacka K, Owczarek K, Caban M, Sosnowska D, Kajszczak D, Lewandowska U. Chemoprotective effects of Japanese quince (Chaenomeles japonica L.) phenol leaf extract on colon cancer cells through the modulation of extracellular signal-regulated kinases/AKT signaling pathway. J Physiol Pharmacol. 2022 Feb;73(1). doi: 10.26402/jpp.2022.1.04. Epub 2022 May 27. PMID: 35639036.

19: Batjargal A, Solek P, Kukula-Koch W, Urjin B, Koch W, Koman D, Dudzinska E. Gurgem-7 toxicity assessment: Regulation of cell survival or death by traditional Mongolian prescription. Ecotoxicol Environ Saf. 2022 Jul 1;239:113660. doi: 10.1016/j.ecoenv.2022.113660. Epub 2022 May 20. PMID: 35605329.

20: Selvaraj J, Vishnupriya V, Sardar H, Balakrishna JP, Rex J, Mohan SK, Vijayalakshmi P, Ponnulakshmi R. Molecular docking analysis of beta-catenin with compounds derived from Lycopersicon esculentum. Bioinformation. 2020 Nov 30;16(11):801-806. doi: 10.6026/97320630016801. PMID: 34803252; PMCID: PMC8573472.

21: Ayan İÇ, Çetinkaya S, Dursun HG, Güneş CE, Şirin S. Anticancer Effect and Phytochemical Profile of the Extract from Achillea ketenoglui against Human Colorectal Cancer Cell Lines. Anticancer Agents Med Chem. 2022;22(9):1769-1779. doi: 10.2174/1871520621666210908110422. PMID: 34503424.

22: Villota H, Moreno-Ceballos M, Santa-González GA, Uribe D, Castañeda ICH, Preciado LM, Pedroza-Díaz J. Biological Impact of Phenolic Compounds from Coffee on Colorectal Cancer. Pharmaceuticals (Basel). 2021 Aug 3;14(8):761. doi:

10.3390/ph14080761. PMID: 34451858; PMCID: PMC8401378.

23: Mondal A, Banerjee S, Bose S, Das PP, Sandberg EN, Atanasov AG, Bishayee A. Cancer Preventive and Therapeutic Potential of Banana and Its Bioactive Constituents: A Systematic, Comprehensive, and Mechanistic Review. Front Oncol. 2021 Jul 7;11:697143. doi: 10.3389/fonc.2021.697143. PMID: 34307163; PMCID: PMC8294041.

24: Çetinkaya S, Çınar Ayan İ, Süntar İ, Dursun HG. The Phytochemical Profile and Biological Activity of <i>Liquidambar orientalis</i> Mill. var. <i>orientalis</i> via NF-κB and Apoptotic Pathways in Human Colorectal Cancer. Nutr Cancer. 2022;74(4):1457-1473. doi: 10.1080/01635581.2021.1952455. Epub 2021 Jul 22. PMID: 34291706.

25: Albogami S, Hassan AM. Assessment of the Efficacy of Olive Leaf (<i>Olea europaea</i> L.) Extracts in the Treatment of Colorectal Cancer and Prostate Cancer Using In Vitro Cell Models. Molecules. 2021 Jul 3;26(13):4069. doi: 10.3390/molecules26134069. PMID: 34279409; PMCID: PMC8272070.

26: Marzo F, Milagro FI, Barrenetxe J, Díaz MT, Martínez JA. Azoxymethane-Induced Colorectal Cancer Mice Treated with a Polyphenol-Rich Apple Extract Show Less Neoplastic Lesions and Signs of Cachexia. Foods. 2021 Apr 15;10(4):863. doi: 10.3390/foods10040863. PMID: 33921048; PMCID: PMC8071383.

27: Villarini M, Acito M, di Vito R, Vannini S, Dominici L, Fatigoni C, Pagiotti R, Moretti M. Pro-Apoptotic Activity of Artichoke Leaf Extracts in Human HT-29 and RKO Colon Cancer Cells. Int J Environ Res Public Health. 2021 Apr 15;18(8):4166. doi: 10.3390/ijerph18084166. PMID: 33920761; PMCID: PMC8071198.

28: Ayouaz S , Oliveira-Alves SC , Serra AT , Lefsih K , Samah M , Bento da Silva A , Madani K , Bronze MR . LC-DAD-ESI-MS/MS analysis and cytotoxic and antiproliferative effects of chlorogenic acid derivative rich extract from Nerium oleander L. pink flowers. Food Funct. 2021 Apr 21;12(8):3624-3634. doi: 10.1039/d0fo02640a. Epub 2021 Mar 31. PMID: 33900304.

29: Castaldo L, Izzo L, Narváez A, Rodríguez-Carrasco Y, Grosso M, Ritieni A. Colon Bioaccessibility under In Vitro Gastrointestinal Digestion of Different Coffee Brews Chemically Profiled through UHPLC-Q-Orbitrap HRMS. Foods. 2021 Jan 17;10(1):179. doi: 10.3390/foods10010179. PMID: 33477307; PMCID: PMC7829986.

30: Yapıcı İ, Altay A, Öztürk Sarıkaya B, Korkmaz M, Atila A, Gülçin İ, Köksal E. In vitro Antioxidant and Cytotoxic Activities of Extracts of Endemic

Tanacetum erzincanense Together with Phenolic Content by LC-ESI-QTOF-MS. Chem Biodivers. 2021 Mar;18(3):e2000812. doi: 10.1002/cbdv.202000812. Epub 2021 Feb 8. PMID: 33464702.

31: Caicedo-Lopez LH, Cuellar-Nuñez ML, Luzardo-Ocampo I, Campos-Vega R, Lóarca-
Piña G. Colonic metabolites from digested <i>Moringa oleifera</i> leaves induced HT-29 cell death via apoptosis, necrosis, and autophagy. Int J Food Sci Nutr. 2021 Jun;72(4):485-498. doi: 10.1080/09637486.2020.1849039. Epub 2020 Dec 10. PMID: 33302731.

32: Chojnacka K, Sosnowska D, Polka D, Owczarek K, Gorlach-Lira K, Oliveira de Verasa B, Lewandowska U. Comparison of phenolic compounds, antioxidant and cytotoxic activity of extracts prepared from Japanese quince (Chaenomeles japonica L.) leaves. J Physiol Pharmacol. 2020 Apr;71(2). doi: 10.26402/jpp.2020.2.05. Epub 2020 Jul 2. PMID: 32633239.

33: Ayouaz S , Oliveira-Alves SC , Lefsih K , Serra AT , Bento da Silva A , Samah M , Karczewski J , Madani K , Bronze MR . Phenolic compounds from Nerium oleander leaves: microwave assisted extraction, characterization, antiproliferative and cytotoxic activities. Food Funct. 2020 Jul 1;11(7):6319-6331. doi: 10.1039/d0fo01180k. Epub 2020 Jul 1. PMID: 32608462.

34: Santana-Gálvez J, Villela-Castrejón J, Serna-Saldívar SO, Cisneros-Zevallos L, Jacobo-Velázquez DA. Synergistic Combinations of Curcumin, Sulforaphane, and Dihydrocaffeic Acid against Human Colon Cancer Cells. Int J Mol Sci. 2020 Apr 28;21(9):3108. doi: 10.3390/ijms21093108. PMID: 32354075; PMCID: PMC7246525.

35: Taha KF, Khalil M, Abubakr MS, Shawky E. Identifying cancer-related molecular targets of Nandina domestica Thunb. by network pharmacology-based analysis in combination with chemical profiling and molecular docking studies. J Ethnopharmacol. 2020 Mar 1;249:112413. doi: 10.1016/j.jep.2019.112413. Epub 2019 Nov 21. PMID: 31760157.

36: Popović BM, Blagojević B, Ždero Pavlović R, Mićić N, Bijelić S, Bogdanović B, Mišan A, Duarte CMM, Serra AT. Comparison between polyphenol profile and bioactive response in blackthorn (Prunus spinosa L.) genotypes from north Serbia-from raw data to PCA analysis. Food Chem. 2020 Jan 1;302:125373. doi: 10.1016/j.foodchem.2019.125373. Epub 2019 Aug 14. PMID: 31442706.

37: Romualdo GR, Rocha AB, Vinken M, Cogliati B, Moreno FS, Chaves MAG, Barbisan LF. Drinking for protection? Epidemiological and experimental evidence on the

beneficial effects of coffee or major coffee compounds against gastrointestinal and liver carcinogenesis. Food Res Int. 2019 Sep;123:567-589. doi: 10.1016/j.foodres.2019.05.029. Epub 2019 May 22. PMID: 31285007.

38: Bułdak RJ, Hejmo T, Osowski M, Bułdak Ł, Kukla M, Polaniak R, Birkner E. The Impact of Coffee and Its Selected Bioactive Compounds on the Development and Progression of Colorectal Cancer In Vivo and In Vitro. Molecules. 2018 Dec 13;23(12):3309. doi: 10.3390/molecules23123309. PMID: 30551667; PMCID: PMC6321559.

39: Dos Reis Luz L, Porto DD, Castro CB, Silva MFS, de Godoy Alves Filho E, Canuto KM, de Brito ES, Becker H, do Ó Pessoa C, Zocolo GJ. Metabolomic profile of Schinopsis brasiliensis via UPLC-QTOF-MS for identification of biomarkers and evaluation of its cytotoxic potential. J Chromatogr B Analyt Technol Biomed Life Sci. 2018 Nov 1;1099:97-109. doi: 10.1016/j.jchromb.2018.09.019. Epub 2018 Sep 18. PMID: 30265941.

40: Agudelo CD, Luzardo-Ocampo I, Campos-Vega R, Loarca-Piña G, Maldonado-Celis ME. Bioaccessibility during In Vitro Digestion and Antiproliferative Effect of Bioactive Compounds from Andean Berry (Vaccinium meridionale Swartz) Juice. J Agric Food Chem. 2018 Jul 18;66(28):7358-7366. doi: 10.1021/acs.jafc.8b01604. Epub 2018 Jul 6. PMID: 29913068.

41: Zhang S, Yang C, Idehen E, Shi L, Lv L, Sang S. Novel Theaflavin-Type Chlorogenic Acid Derivatives Identified in Black Tea. J Agric Food Chem. 2018 Apr 4;66(13):3402-3407. doi: 10.1021/acs.jafc.7b06044. Epub 2018 Mar 21. PMID: 29534564.

42: Cuellar-Nuñez ML, Luzardo-Ocampo I, Campos-Vega R, Gallegos-Corona MA, González de Mejía E, Loarca-Piña G. Physicochemical and nutraceutical properties of moringa (Moringa oleifera) leaves and their effects in an in vivo AOM/DSS-induced colorectal carcinogenesis model. Food Res Int. 2018 Mar;105:159-168. doi: 10.1016/j.foodres.2017.11.004. Epub 2017 Nov 6. PMID: 29433203.

43: Vancsik T, Kovago C, Kiss E, Papp E, Forika G, Benyo Z, Meggyeshazi N, Krenacs T. Modulated electro-hyperthermia induced loco-regional and systemic tumor destruction in colorectal cancer allografts. J Cancer. 2018 Jan 1;9(1):41-53. doi: 10.7150/jca.21520. PMID: 29290768; PMCID: PMC5743710.

44: Nam SH, Ko JA, Jun W, Wee YJ, Walsh MK, Yang KY, Choi JH, Eun JB, Choi J, Kim YM, Han S, Nguyen TTH, Kim D. Enzymatic synthesis of chlorogenic acid glucoside using dextransucrase and its physical and functional properties.

Enzyme Microb Technol. 2017 Dec;107:15-21. doi: 10.1016/j.enzmictec.2017.07.011. Epub 2017 Jul 29. PMID: 28899482.

45: Martinez-Saez N, Hochkogler CM, Somoza V, Del Castillo MD. Biscuits with No Added Sugar Containing Stevia, Coffee Fibre and Fructooligosaccharides Modifies α-Glucosidase Activity and the Release of GLP-1 from HuTu-80 Cells and Serotonin from Caco-2 Cells after In Vitro Digestion. Nutrients. 2017 Jul 4;9(7):694. doi: 10.3390/nu9070694. PMID: 28677657; PMCID: PMC5537809.

46: Ombra MN, d'Acierno A, Nazzaro F, Riccardi R, Spigno P, Zaccardelli M, Pane C, Maione M, Fratianni F. Phenolic Composition and Antioxidant and Antiproliferative Activities of the Extracts of Twelve Common Bean (<i>Phaseolus vulgaris</i> L.) Endemic Ecotypes of Southern Italy before and after Cooking. Oxid Med Cell Longev. 2016;2016:1398298. doi: 10.1155/2016/1398298. Epub 2016 Dec 25. PMID: 28105248; PMCID: PMC5220516.

47: Banerjee N, Kim H, Talcott ST, Turner ND, Byrne DH, Mertens-Talcott SU. Plum polyphenols inhibit colorectal aberrant crypt foci formation in rats: potential role of the miR-143/protein kinase B/mammalian target of rapamycin axis. Nutr Res. 2016 Oct;36(10):1105-1113. doi: 10.1016/j.nutres.2016.06.008. Epub 2016 Jun 14. PMID: 27865352.

48: Hou N, Liu N, Han J, Yan Y, Li J. Chlorogenic acid induces reactive oxygen species generation and inhibits the viability of human colon cancer cells. Anticancer Drugs. 2017 Jan;28(1):59-65. doi: 10.1097/CAD.0000000000000430. PMID: 27603595.

49: Lee J, Kim YS, Lee J, Heo SC, Lee KL, Choi SW, Kim Y. Walnut Phenolic Extract and Its Bioactive Compounds Suppress Colon Cancer Cell Growth by Regulating Colon Cancer Stemness. Nutrients. 2016 Jul 21;8(7):439. doi: 10.3390/nu8070439. PMID: 27455311; PMCID: PMC4963915.

50: Mira A, Shimizu K. In vitro Cytotoxic Activities and Molecular Mechanisms of Angelica shikokiana Extract and its Isolated Compounds. Pharmacogn Mag. 2015 Oct;11(Suppl 4):S564-9. doi: 10.4103/0973-1296.172962. PMID: 27013795; PMCID: PMC4787089.

51: Van Hecke T, Wouters A, Rombouts C, Izzati T, Berardo A, Vossen E, Claeys E, Van Camp J, Raes K, Vanhaecke L, Peeters M, De Vos WH, De Smet S. Reducing Compounds Equivocally Influence Oxidation during Digestion of a High-Fat Beef Product, which Promotes Cytotoxicity in Colorectal Carcinoma Cell Lines. J Agric Food Chem. 2016 Feb 24;64(7):1600-9. doi: 10.1021/acs.jafc.5b05915. Epub 2016

Feb 12. PMID: 26836477.

52: Choi DW, Lim MS, Lee JW, Chun W, Lee SH, Nam YH, Park JM, Choi DH, Kang CD, Lee SJ, Park SC. The Cytotoxicity of Kahweol in HT-29 Human Colorectal Cancer Cells Is Mediated by Apoptosis and Suppression of Heat Shock Protein 70 Expression. Biomol Ther (Seoul). 2015 Mar;23(2):128-33. doi: 10.4062/biomolther.2014.133. Epub 2015 Mar 1. PMID: 25767680; PMCID: PMC4354313.
